# Can harmonisation of outcomes bridge the translation gap for pre-clinical research? A systematic review of outcomes measured in mouse models of type 2 diabetes

**DOI:** 10.1186/s12967-020-02649-6

**Published:** 2020-12-09

**Authors:** Nicola L. Harman, Adrián Sanz-Moreno, Stamatia Papoutsopoulou, Katie A. Lloyd, Kamar E. Ameen-Ali, Malcolm Macleod, Paula R. Williamson

**Affiliations:** 1 Department of Health Data Science, University of Liverpool, Liverpool, L69 3GL UK; 2 German Mouse Clinic, Institute of Experimental Genetics, HMGU, Neuherberg, 85764 Germany; 3 Cellular and Molecular Physiology, Institute of Translational Medicine, University of Liverpool, Liverpool, L69 3GL UK; 4 Clinical Translational Research Innovation Centre (CTRIC), Altnagelvin Hospital, University of Ulster, Londonderry, BT47 6SB UK; 5 Translational and Clinical Research Institute, Faculty of Medical Sciences, Newcastle University, Newcastle, NE4 5PL UK; 6 Centre for Clinical Brain Sciences, University of Edinburgh, Edinburgh, UK

**Keywords:** Type 2 diabetes, Pre-clinical research, Mouse models of type 2 diabetes, Core outcome set, Translational research, Clinical research, Pre-clinical systematic review

## Abstract

**Background:**

In pre-clinical research, systematic reviews have the potential to mitigate translational challenges by facilitating understanding of how pre-clinical studies can inform future clinical research. Yet their conduct is encumbered by heterogeneity in the outcomes measured and reported, and those outcomes may not always relate to the most clinically important outcomes. We aimed to systematically review outcomes measured and reported in pre-clinical in vivo studies of pharmacological interventions to treat high blood glucose in mouse models of type 2 diabetes.

**Methods:**

A systematic review of pre-clinical in vivo studies of pharmacological interventions aimed at addressing elevated blood glucose in mouse models of type 2 diabetes was completed. Studies were screened for eligibility and outcomes extracted from the included studies. The outcomes were recorded verbatim and classified into outcome domains using an existing outcome taxonomy. Outcomes were also compared to those identified in a systematic review of registered phase 3/4 clinical trials for glucose lowering interventions in people with type 2 diabetes.

**Results:**

Review of 280 included studies identified 532 unique outcomes across 19 domains. No single outcome, or domain, was measured in all studies and only 132 (21%) had also been measured in registered phase 3/4 clinical trials. A core outcome set, representing the minimum that should be measured and reported, developed for type 2 diabetes effectiveness clinical trials includes 18 core outcomes, of these 12 (71%) outcomes were measured and reported in one or more of the included pre-clinical studies.

**Conclusions:**

There is heterogeneity of outcomes reported in pre-clinical research. Harmonisation of outcomes across the research pathway using a core outcome set may facilitate interpretation, evidence synthesis and translational success, and may contribute to the refinement of the use of animals in research.

*Systematic review registration*: The study was prospectively registered on the PROSPERO Database, registration number CRD42018106831

## Background

Clinical trials are undertaken to evaluate the effectiveness and safety of treatments in defined populations, and use pre-defined outcome measures. However, there is often marked variability between trials in the outcomes measured and reported which contributes to research waste through the inability to compare findings and synthesise evidence from multiple trials [1]. These issues can be addressed through the use of a core outcome set (COS), defined as “the minimum [set of outcomes] that should be measured and reported in all clinical trials of a specific condition” [2]. Indeed, in the case of rheumatoid arthritis, a core outcome set has increased the consistency of outcome reporting, and use of the COS has increased over time [3]. There are currently 337 COSs spanning 31 disease areas that have been developed for clinical research or practice or both [4]. However, despite the uptake of COSs in clinical trials, little is known about their relationship to the outcomes measured at other stages of the research pathway.

In pre-clinical research there is a recognised issue in the ability of animal models to predict effectiveness in humans, with large variability in translational success rates [5–7]. Pre-clinical systematic reviews have been proposed as a way to improve understanding of pre-clinical effectiveness and how this can inform clinical trials [8–10]. Yet, as in clinical trials, the ability to systematically review the literature is impacted by issues of methodological rigour and further compounded by heterogeneity in the outcomes measured and reported [1, 11]. Initiatives to improve the reporting of methodological details, for instance, the Animal Research: Reporting In Vivo Experiments (ARRIVE) guidelines [12], do not consider the choice of study outcome(s), that may impact not only on the ability to systematically compare and contrast the study results but also on the translatability of pre-clinical research to later phase trials. While the STAIR criteria for preclinical stroke research does address this issue, their requirements are rather broad [13]. We sought to explore the issue of outcome heterogeneity in pre-clinical research and the potential application of a COS using type 2 diabetes research as a case study. Type 2 diabetes is a global health concern; it has been estimated that 700 million people aged 20–79 will be affected by diabetes by 2045, the majority of these cases being type 2 diabetes [14–16]. There are a number of established animal models for the study of type 2 diabetes [17, 18] with mouse models offering several advantages, including ease of induction of type 2 diabetes, a relatively short breeding span, and availability of physiological and invasive testing [19]. Mice are widely used in endocrine and metabolic research and, if all research areas are taken into account, represent the most widely used animal model in pre-clinical research [20]. We aimed to systematically review outcomes measured in pre-clinical research for type 2 diabetes using a mouse model, and to compare these to outcomes measured in clinical trials of glucose lowering interventions in type 2 diabetes [21]. Finally we examine the extent of the applicability of an existing COS [22] for type 2 diabetes in pre-clinical research.

## Methods

### Search strategy

Relevant pre-clinical animal studies were identified with a combined search of MEDLINE, PubMED and SCOPUS using search terms specific to each database (Table 1). Searches were undertaken on the 16th July 2018.

**Table 1:** Search strategies

MEDLINE	
Line	Search term
1	Exp models,animal/
2	Exp animals/
3	Exp humans/
4	1 OR 2
5	4 NOT 3
6	Mouse ab.ti.tw
7	5 AND 6
8	Pyrazines/or Glucagon-Like Peptide 1/or Adamantane/or Blood Glucose/or exp Hypoglycemic Agents/or Pyrrolidines/or Dipeptidyl-Peptidase IV Inhibitors/or Dipeptidyl Peptidase 4/or Diabetes Mellitus, Type 2/or DPP-4.mp. or Triazoles/
9	7 AND 8
Pub Med	
1	Exp models,animal/
2	Exp humans/
3	1 NOT 2
4	Mouse ab.ti.tw
5	3 AND 4
6	Pyrazines/or Glucagon-Like Peptide 1/or Adamantane/or Blood Glucose/or exp Hypoglycemic Agents/or Pyrrolidines/or Dipeptidyl-Peptidase IV Inhibitors/or Dipeptidyl Peptidase 4/or Diabetes Mellitus, Type 2/or DPP-4 or Triazoles/
7	5 AND 6
SCOPUS	
(((TITLE-ABS-KEY (mouse) AND TITLE-ABS-KEY (glucose))) AND (TITLE-ABS-KEY (diabetes))) AND (INDEXTERMS (nonhuman)) AND (LIMIT-TO (SUBJAREA, “PHAR”))	

Returned entries were exported to Endnote, screened for duplicates and then uploaded into the CAMARADES-NC3Rs Preclinical Systematic Review & Meta-analysis Facility (SYRF) (www.syrf.org.uk, accessed 3rd March-2020) for screening.

The database held on www.preclinicaltrials.eu was also searched for ongoing registered trials of glucose lowering interventions for diabetes, but none were identified (July 2018).

The study protocol, including the search strategy, was prospectively registered on the PROSPERO international prospective register of systematic reviews (https://www.crd.york.ac.uk/prospero/display_record.php?ID=CRD42018106831).

### Eligibility criteria

Publications reporting a pharmacological intervention aimed at lowering blood glucose in a mouse model for type 2 diabetes were eligible for inclusion. Eligible mouse models included dietary induced, chemically induced, monogenic or polygenic models. To be eligible for inclusion, studies must have been undertaken in the context of type 2 diabetes and not solely in other related metabolic disorders, for example, metabolic syndrome, obesity or insulin resistance. There were no restrictions on the year of publication.

Studies were excluded if they met any of the following exclusion criteria: publications reporting the use of mouse models in other related metabolic disorders but not in type 2 diabetes; publications focusing on the prevention of type 2 diabetes only; publications reporting interventions in other animal models; publications reporting in vitro studies only; publications reporting non-pharmacological interventions for type 2 diabetes; publications primarily focused on interventions for complications of type 2 diabetes (e.g. retinopathy, neuropathy, cardiovascular disease, gastroparesis); publications reporting interventions exclusively for type 1 diabetes or gestational diabetes; studies that are solely mechanistic. Publications were also excluded if they used a mouse model inappropriate for the study of type 2 diabetes including, but not limited to, non obese diabetic mice, Akita mice, viral induced diabetes, alloxan induced diabetes, high dose streptozotocin (100–200 mg/kg). Streptozotocin models were included if a low dose was used to induce diabetes and the study specified the use of the model in the context of type 2 diabetes.

### Assessment of study eligibility

Abstracts were reviewed in duplicate by a team of reviewers (NH, AS-M, SP, KL and KAA). Due to the large number of included abstracts, a 25% sample (in 10-year blocks) was taken forward to full text review. At full text review, a 10% duplicate screening batch check was completed for each reviewer before proceeding with single review. Where disagreement or uncertainty about inclusion of a study was noted, the reviewers discussed the study before reaching a decision. No study required third reviewer arbitration.

### Data extraction

Data extraction from included full texts was undertaken by NH. Data on the year of publication, region of work and the mouse model used was extracted along with the outcomes measured. Data on outcomes was extracted from the methods and results sections of papers along with figures, tables and Additional file 1 where available. In cases of composite outcomes, all component outcomes were extracted. Where data on a specific adverse event was collected the outcome was listed twice, once as an adverse event and once as the specific outcome.

### Outcome classification

Each outcome was reviewed and grouped with other outcomes if they measured the same aspect albeit using a different method. Each outcome was categorised according to the COMET taxonomy [23]. This taxonomy comprises 38 domains under five areas (death, physiological/clinical, life impact, resource use and adverse events). Outcome grouping and categorisation was cross checked by AS-M.

### Comparison with clinical trials

Outcomes were then compared to those identified from a previous review of phase 3/4 registered clinical trials [21] and to those included in the core outcome set for type 2 diabetes [22].

## Results

### Characteristics of included studies

**Fig. 1:**
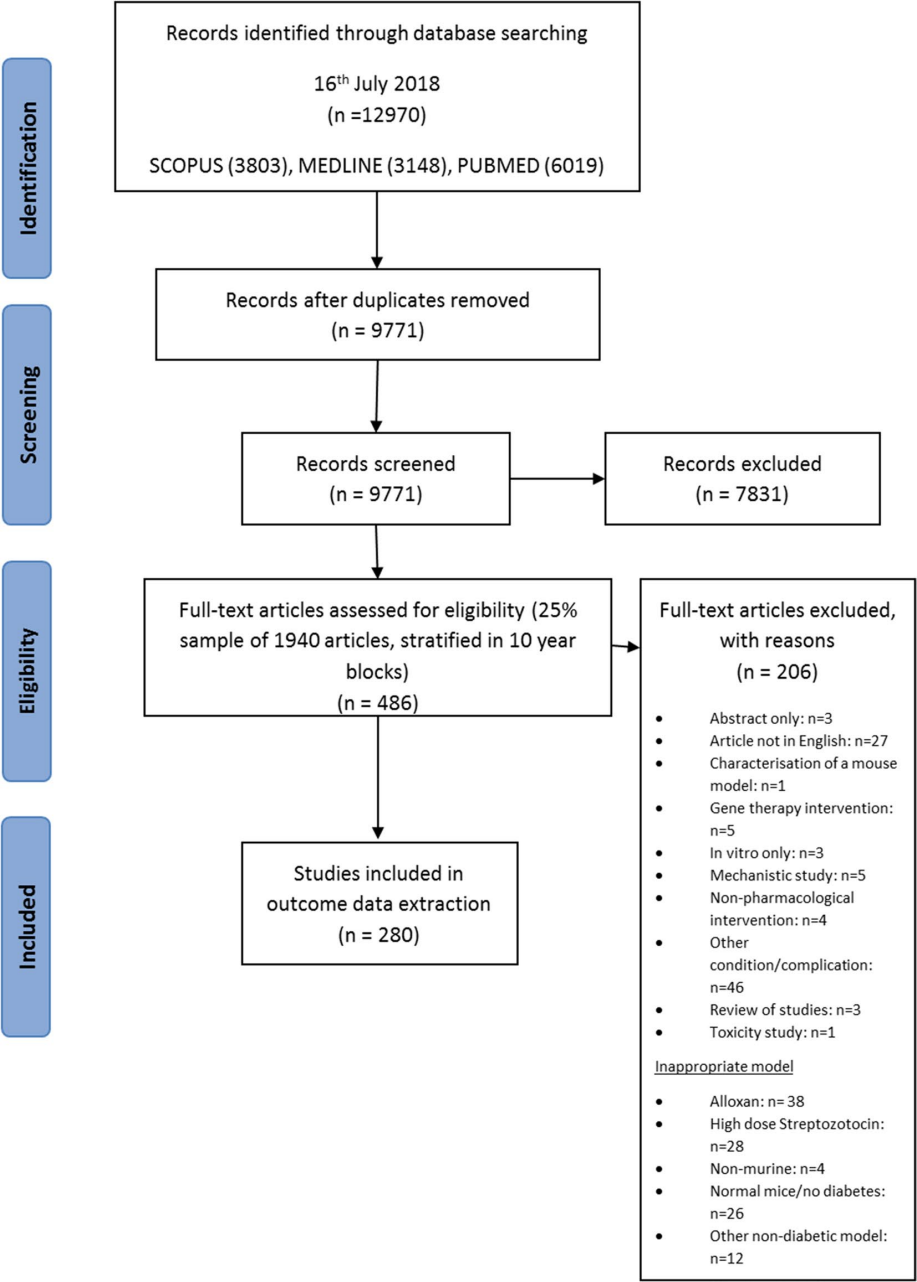
PRISMA flow diagram

A systematic review was performed, to identify relevant pre-clinical in vivo studies using a mouse model of type 2 diabetes. A sample of 25% of included abstracts was assessed for eligibility at the full text stage and outcomes extracted from 280 eligible studies (Fig. 1). All studies used a mouse model of type 2 diabetes with the majority (63%) using a genetic model, for example, KK-Ay or Lepr ^db/db^mice. The characteristics of the included studies are described in Table 2. A full list of the included studies is available in Additional file 1-included studies.

**Table 2:** Description of included studies

	N (%)
Year	
1980–1989	8 (3)
1990–1999	23 (8)
2000–2009	89 (32)
2010–2018	160 (57)
Region of work	
Africa	5 (2)
Asia	142 (51)
Europe	76 (27)
North America	55 (20)
South America	2 (1)
Mouse model	
Chemically induced diabetes	38 (14)
Diet induced diabetes	39 (14)
Genetic model	176 (63)
Mixed models–chemical and diet	12 (4)
Mixed models–chemical and genetic	3 (1)
Mixed models–diet and genetic	12 (4)

### Outcomes measured in pre-clinical studies

A total of 2874 individual outcomes were extracted with a median of 8 outcomes per trial (range 1–46). Each outcome was reviewed and categorised using the COMET taxonomy [23] (Table 3). Outcomes were also tagged if they had also been measured in phase 3/4 clinical trials in Type 2 diabetes identified in a previous systematic review [21].

**Table 3:** Comparison of pre-clinical and clinical trial outcomes by domain

Outcome domain	Pre-clinical in vivo mouse studies		Phase 3/4 clinical trials [20]	
	Total number of unique outcomes within domain (verbatim outcomes)	Number of studies reporting one or more outcomes in the domain (%)	Total number of unique outcomes within domain (verbatim outcomes)	Number of studies reporting one or more outcomes in the domain (%)
1. Mortality/survival	2 (7)	7 (2.5)	2 (3)	3 (2.2)
2. Blood and lymphatic system outcomes	12 (23)	13 (4.6)	14 (19)	9 (6.5)
3. Cardiac outcomes	6 (11)	8 (2.9)	11 (56)	20 (14.5)
4. Congenital, familial and genetic outcomes	0 (0)	0 (0)	0 (0)	0 (0)
5. Endocrine outcomes	32 (324)	164 (58.6)	14 (50)	31 (22.5)
6. Ear and labyrinth outcomes	0 (0)	0 (0)	0 (0)	0 (0)
7. Eye outcomes	0 (0)	0 (0)	2 (2)	2 (1.4)
8. Gastrointestinal outcomes	7 (9)	7 (2.5)	12 (20)	5 (3.6)
9. General outcomes	26 (296)	171 (61.1)	40 (146)	65 (47.1)
10. Hepatobiliary outcomes	23 (123)	70 (25)	12 (25)	12 (8.7)
11. Immune system outcomes	35 (84)	26 (9.3)	32 (73)	28 (20.3)
12. Infection and infestation outcomes	0 (0)	0 (0)	7 (8)	4 (2.9)
13. Injury and poisoning outcomes	0 (0)	0 (0)	0 (0)	0 (0)
14. Metabolism and nutrition outcomes	317 (1816)	279 (99.6)	105 (582)	121 (87.7)
15. Musculoskeletal and connective tissue outcomes	21 (42)	19 (6.8)	2 (2)	2 (1.4)
16. Outcomes relating to neoplasms: benign, malignant and unspecified (including cysts and polyps)	0 (0)	0 (0)	0 (0)	0 (0)
17. Nervous system outcomes	14 (18)	7 (2.5)	16 (16)	6 (4.3)
18. Pregnancy, puerperium and perinatal outcomes	0 (0)	0 (0)	0 (0)	0 (0)
19. Renal and urinary outcomes	19 (45)	30 (10.7)	53 (76)	27 (19.6)
20. Reproductive system and breast outcomes	0 (0)	0 (0)	0 (0)	0 (0)
21. Psychiatric outcomes	2 (3)	3 (1.1)	2 (2)	2 (1.4)
22. Respiratory, thoracic and mediastinal outcomes	2 (5)	4 (1.4)	3 (11)	23 (16.7)
23. Skin and subcutaneous tissue outcomes	0 (0)	0 (0)	1 (1)	1 (0.7)
24. Vascular outcomes	8 (9)	8 (2.9)	56 (134)	52 (37.7)
25. Physical functioning	4 (27)	17 (6.1)	3 (7)	5 (3.6)
26. Social functioning	0 (0)	0 (0)	4 (6)	5 (3.6)
27. Role functioning	0 (0)	0 (0)	3 (6)	3 (2.2)
28. Emotional functioning/wellbeing	0 (0)	0 (0)	23 (28)	8 (5.8)
29. Cognitive functioning	1 (2)	2 (0.7)	16 (22)	2 (1.4)
30. Global quality of life	0 (0)	0 (0)	2 (5)	4 (2.9)
31. Perceived health status	0 (0)	0 (0)	2 (4)	4 (2.9)
32. Delivery of care	1 (1)	1 (0.4)	18 (60)	30 (21.7)
33. Personal circumstances	0 (0)	0 (0)	0 (0)	0 (0)
34. Economic	0 (0)	0 (0)	6 (6)	4 (2.9)
35. Hospital	0 (0)	0 (0)	3 (4)	3 (2.2)
36. Need for intervention	0 (0)	0 (0)	7 (24)	16 (11.6)
37. Societal/carer burden	0 (0)	0 (0)	0 (0)	0 (0)
38. Adverse events/effects	5 (29)	20 (7.1)	4 (46)	33 (23.9)

Some outcomes have been coded twice based on the context of measurement. Specifically total protein has been coded as ‘general outcomes’ and, where the reason for measurement was specified, this has been coded as ‘renal and urinary outcomes’

One study measured alkaline phosphatase (ALP), aspartate aminotransferase (AST) and alanine aminotransferase (ALT) specifically in the context of renal function and so these outcomes have been coded in both the ‘hepatobiliary outcomes’ domain and for one study coded in the ‘renal and urinary outcomes’ domain

Phosphorylated c-Jun N-terminal kinase (p-JNK) expression has been coded as ‘general outcomes’ and also for one study as ‘endocrine’ where this was measured specifically in relation to pancreatic fibrosis

The 2874 outcomes represented 532 unique outcomes across 19 domains. Of the unique outcomes, 205 (39%) represented outcomes relevant to the mechanism of drug action rather than safety or efficacy. No single outcome was measured in all studies. The most frequently represented domain was “metabolism and nutrition” with all but one study (279/280) measuring one or more outcomes within the domain. Within this domain, 90% (253/279) measured blood or plasma glucose or both; and 99% (277/279) either blood/plasma glucose, tissue glucose, glycaemic control, glucose tolerance, hypoglycaemia or urinary glucose. Also within the “metabolism and nutrition” domain just under half of studies (44%) measured one or more lipid or lipoprotein markers of cardiovascular disease risk. Emerging cardiovascular risk markers such as biomarkers of oxidative stress were less frequently measured (9% of studies). 171 of 280 studies (61%) reported “general outcomes” (not attributed to a certain body system), for example, outcomes relating to body weight or composition (166/280, 59%).164 of 280 studies (59%) included an outcome in the “endocrine outcomes” domain and of these 151 (92%) included an outcome relating to insulin, c-peptide or glucagon. Adverse events or effects were less frequently reported with only 7% of trials including one or more outcomes in this domain.

### Comparison with outcomes measured in later phase clinical trials

All domains measured in pre-clinical in vivo studies had also been measured in phase 3/4 clinical trials. The distribution of outcomes across the COMET taxonomy domains was similar between pre-clinical and clinical studies with the exception of “vascular”, “cardiac”, “adverse events” and “delivery of care” outcomes that were more prevalent in phase 3/4 trials; and “endocrine outcomes”, that were more frequently measured pre-clinically. The clinical trials also included outcomes in an additional 11 domains (Table 3). Of these additional domains, “economic”, “hospital”, “role functioning” and “perceived health status” could only relate to human intervention studies.

Importantly, of the 532 unique outcomes reported pre-clinically, only 21% had also been measured in type 2 diabetes clinical trials. This may reflect the prevalence of mechanistic outcomes in pre-clinical studies, or greater feasibility for measuring certain outcomes in the pre-clinical setting compared with clinical trials.

### Comparison with an existing core outcome set

**Fig. 2:**
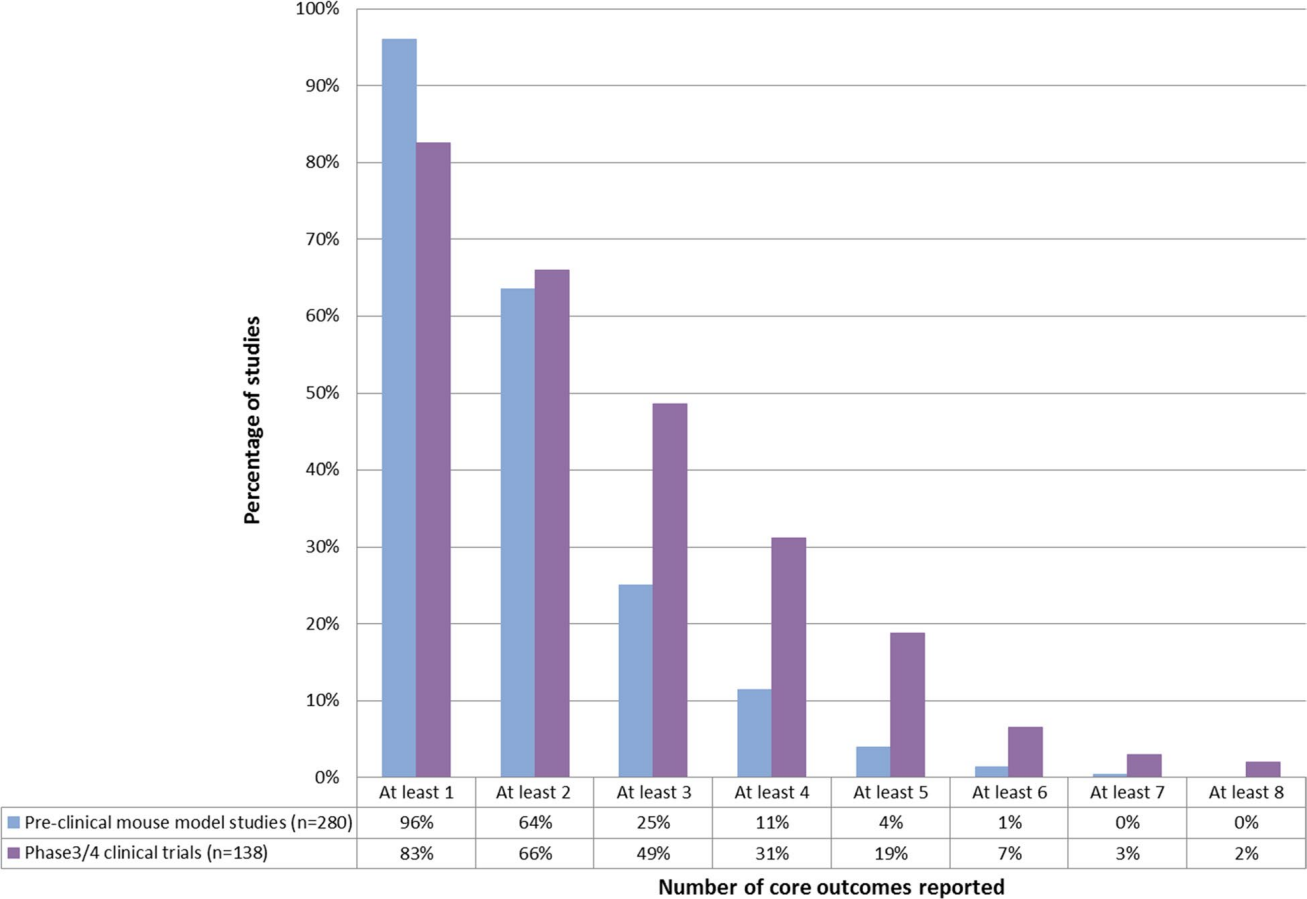
Proportion of pre-clinical and clinical studies measuring one or more of the core outcomes

Core outcome sets (COS) represent the minimum set of outcomes that should be measured and reported in every clinical trial of a specific area of health [24]. Their purpose is to reduce the heterogeneity in outcomes measured in clinical trials of a particular condition, facilitate evidence synthesis, and promote the measurement of outcomes relevant to all stakeholders. A COS for glucose lowering interventions for type 2 diabetes has been developed [22] and the outcomes measured pre-clinically were compared to this. The core outcome set includes 18 outcomes, and of these 17 could potentially be measured in a mouse model. Twelve (71%) of these core outcomes were represented to some extent in the outcomes measured in pre-clinical studies (Table 4). However, studies typically measured only 1 or 2 outcomes and no single study reported more than seven outcomes in the COS (Fig. 2). Similar patterns were observed in clinical trials, registered prior to the publication of the COS, although a larger proportion of trials measured multiple core outcomes. In pre-clinical studies there were multiple outcomes reported that could be used to measure a core outcome (Table 4). Furthermore within these there were multiple methods of assessment. For example, “glycaemic control” was reported in 40 studies using four different outcomes of which glycated haemoglobin (HbA1c) was the most frequently measured (35/40 studies). There are four, commonly used, methods for the measurement of HbA1c [25], details of the method used were reported in 29/35 papers. Each of the four methods was used at least once with immunoassay used most frequently (n = 18) followed by, ion-exchange high-performance liquid chromatography (HPLC) (n = 4), boronate affinity HPLC (n = 6), and enzymatic assays (n = 1), highlighting the variability in “how” outcomes are measured.

**Table 4:** Comparison of the core outcome set for clinical trials in type 2 diabetes and outcomes in pre-clinical studies

Core outcome	Domain	Outcomes reported in pre-clinical in vivo studies	Number of pre-clinical studies reporting the outcome (%)	Number of phase 3/4 clinical trials reporting the outcome (%)
Overall survival—how long someone lives	Death	Mortality, survival	7 (2.5)	2 (1.4)^a^
Death from a diabetes related cause such as heart disease	Death	No study specifically described death from a diabetes related cause	0 (0)	1 (0.7)
Glycaemic control—how well someone’s blood glucose is controlled	Physiological/clinical	Glycated haemoglobin (HbA1c), glycated serum proteins, euglycaemic duration, fructosamine	40 (14.3)	93 (67.4)
Body weight—how much someone weighs	Physiological/clinical	Body weight	165 (58.9)	54 (39.1)
Kidney function—how well someone’s kidneys are working	Physiological/clinical	Blood urea nitrogen (BUN), creatinine, electrolytes, renal fructose, renal sorbitol, total protein, urine volume, kidney function^b^	23 (8.2)	25 (18.1)
Hyperglycaemia—how often someone has high blood glucose	Physiological/clinical	Blood glucose	253 (90.4)	64 (46.4)
Hypoglycaemia—how often someone has low blood glucose levels	Physiological/clinical	Hypoglycaemic and hypoglycaemic duration^c^	3 (1.1)	45 (32.6)
Visual deterioration or blindness—if someone’s eyesight gets worse or if they have loss of vision including blindness	Physiological/clinical	No studies measured this outcome	0 (0)	2 (1.4)
Neuropathy—damage to the nerves caused by high glucose. This can lead to tingling and pain or numbness in the feet or legs. It can also affect bowel control; stomach emptying and sexual function	Physiological/clinical	Hyperalgesia, long term potentiation, pain threshold	5 (1.8)	2 (1.4)
Having gangrene or having an amputation of the leg, foot or took	Physiological/clinical	No studies measured this outcome	0 (0)	0 (0)
Nonfatal myocardial infarction—having a heart attack that is not fatal	Physiological/clinical	No studies measured this outcome	0 (0)	2 (1.4)
Heart failure	Physiological/clinical	Heart weight, appearance of the heart, heart histology	6 (2.1)	11 (7.9)
Cerebrovascular disease—including stroke, subarachnoid haemorrhage, transient ischaemic attack and vascular dementia	Physiological/clinical	No studies measured this outcome	0 (0)	0 (0)
Hyperglycaemic emergencies (to include diabetic ketoacidosis and hyperosmolar hyperglycaemic state)	Physiological/clinical	Ketone bodies, lactate, β-hydroxybutyrate	8 (2.9)	11 (7.9)
Global quality of life—someone’s overall quality of life including physical, mental and social wellbeing	Life impact	Object recognition, anxiety, mental status, food or water intake^d^	111 (39.6)	7 (5.1)
		Object recognition	2 (0.7)	–
		Anxiety/mental status	3 (1.1)	–
		Food or water intake	110 (39.3)	–
Activities of daily living - being able to complete usual everyday tasks and activities including those related to personal care; house hold tasks or community based tasks	Life impact	Behaviour, locomotor activity, open field assessment, exploratory activity, ambulatory activity, physical activity, external appearance, coat fur, hair colour	18 (6.4)	2 (1.4)
How often someone is admitted to hospital because of their diabetes	Resource use	Not applicable	N/A	3 (2.2)
Side effects of treatment- any unwanted effects of the treatment	Adverse events	Organ toxicity, adverse effects, side effects, toxicity	20 (7.1)	34 (26.6)

^a^In clinical trials no reports of death would often be assumed to mean no deaths even if the number of deaths as “zero” is not explicitly stated

^b^Alanine aminotransferase (ALT), alkaline phosphatase (ALP) and aspartate aminotransferase (AST) were measured in one study in relation to kidney function. However, these are generally used as biomarkers for liver function and so have not been included

^c^Blood glucose may also indicate hypoglycaemia, this has been reported separately

^d^Body weight may also represent quality of life but this has been reported as a separate outcome

Outcomes in the COS that were not reported, or infrequently reported, represented longer term outcomes associated with morbidities (nephropathy, neuropathy, retinopathy, cardiovascular and cerebrovascular disease) resulting from long term insulin resistance [26]. Measuring such complications in otherwise healthy, usually adolescent laboratory mice where the rates of spontaneous development of these complications of diabetes is low would bring some challenges, including the ethical and resource costs of the longer duration of experiments which would be required.

## Discussion

Addressing methodological issues in pre-clinical research will help researchers take one step closer to achieving successful translation of safe and effective treatments [12, 27, 28]. However, the issue of outcome heterogeneity in pre-clinical studies, demonstrated here for type 2 diabetes, has been overlooked, impacting on the ability to synthesise evidence, contributing to research waste and widening the translational gap.

Initiatives to harmonise outcomes have focused on later phase effectiveness trials [2, 24] yet there is potential to apply COS across the research pathway. In the case of type 2 diabetes, over 70% of the existing COS was measured, to some extent, in publications using pre-clinical mouse models. Discordance was observed for outcomes relating to long term complications but these too were infrequently measured in clinical trials with “gangrene and amputation of the leg, foot or toe”, and “cerebrovascular disease” not measured at all and “deterioration of vision” and “myocardial infarction” each measured in a single clinical trial [21]. There were also limits of the pre-clinical search strategy which excluded studies that used specific mouse models of a long term diabetes complication. Mice display a different rate of development/aging to humans that cannot easily be converted between species [29, 30]; consequently, disease progression and time to the onset of long term complications may not be feasible to assess unless a specific animal model, pre-disposed to the development of such conditions, is used.

Whilst mouse models are the most frequently used pre-clinical animal model, there are limitations in the physiological assessments that can be undertaken. A review of large animal and non-human primate models may identify further overlap of outcomes with those assessed in phase 3/4 clinical trials due to the ability to perform particular physiological assessments in these larger animals.

Death is reported in clinical trials, either as a specific outcome or in the collection of serious adverse events, and is routinely recorded in clinical practice but in the pre-clinical setting it is widely accepted that “death as an endpoint to a procedure should be avoided as far as possible and replaced by earlier, humane endpoints” [31]. Instead, alternative surrogate outcomes for death may be more appropriate and alleviate terminal distress in mice whilst also capturing the core outcome [32, 33.]

In the present study we have applied surrogate markers of quality of life including “food and water intake”, measured in 39% of studies. Yet in these studies the reason for measurement was not specified and, in the case of some diabetes treatments, may indicate assessment of a side effect of treatment (weight gain) or polydipsia (a symptom of elevated blood glucose). Animal welfare encompasses an animal’s overall quality of life, taking into consideration its physical and psychological health along with the suitability of living conditions that give the animal opportunity to exhibit natural behaviours. Assessment of welfare is a critical component of research involving animals but this may go unreported in study publications. This under-reporting is further compounded by multiple methods of assessment and clarity is needed on how quality of life should be measured [34].

A COS has the potential to reduce the risk of outcome reporting bias, an issue that has been highlighted in both pre-clinical and clinical research [35, 36]. For a COS to contribute to reducing the risk of reporting bias there is an expectation that it is used in its entirety or that clear justification is made for why some outcomes have not been measured. It is important to recognise that it may not be practical, or indeed ethical, to measure the full set of core outcomes in every pre-clinical study and instead data on the core outcomes may be collected by the triangulation of data from multiple pre-clinical studies. Using glycaemic control as an example in the present study it is clear that not only are there different ways to define the outcome but, in the case of HbA1c, multiple methods of measurement. For pre-clinical studies to apply the COS there needs to be clear reporting on which of the outcomes will be measured, including reasons why outcomes are not assessed, together with consensus on “how” each of the core outcome should be measured and further work is warranted in this area.

## Conclusion

The COS developed for type 2 diabetes shows a large overlap with outcomes already measured and reported in pre-clinical research using a mouse model of type 2 diabetes. Application of the COS, using agreed methods, in both pre-clinical research and clinical trials will mean that the same outcomes are measured and reported as a minimum, across the research pathway, facilitating evidence synthesis that has the potential to identify the most promising treatments.

## Supplementary information

**Additional file 1.** Included Studies

## Data Availability

The datasets used and/or analysed during the current study are available from the corresponding author on reasonable request. A full list of included studies used to identify outcomes is provided in Additional file 1-included studies.

## Abbreviation

COS: Core outcome set
